# Interval-compressed chemotherapy in patients with Ewing’s sarcoma: report on feasibility and toxicity from a North African center

**DOI:** 10.3389/fonc.2026.1710836

**Published:** 2026-04-23

**Authors:** Nesrine Kooli, Hajer Ben Mansour, Yosr Zenzri, Yoldez Houcine, Amina Mokrani, Khedija Meddeb, Salma Kamoun, Mouna Ayadi, Nesrine Chraiet, Henda Rais

**Affiliations:** 1 Medical Oncology Department, Institut Salah Azaiz, Tunis, Tunisia; 2 Medical Faculty of Tunis, Tunis El Manar University, Tunis, Tunisia; 3 Pathology Department B, Institut Salah Azaiz, Tunis, Tunisia

**Keywords:** cancer chemotherapy protocols, drug toxicity and adverse effect, Ewing’s sarcoma, LMIC (low- and middle-income countries), sarcoma treatment

## Abstract

**Background:**

Ewing’s sarcoma (ES) is the second most common malignant bone tumor in patients under 20 years of age. The “Euro-Ewing 2012” trial established induction therapy with the 2-week-interval VDC/IE (Vincristine-Doxorubicine-Cyclophosphamide/Ifosfamide-Etoposide) as the standard of care. However, real-world data remain limited. We aimed to determine the feasibility, tolerance, and effectiveness of this protocol.

**Methods:**

We conducted a retrospective, single-institutional cohort study, including patients with ES treated between January 2018 and April 2024 using the interval-compressed VDC/IE protocol. Data were collected on feasibility, toxicity, and outcomes.

**Results:**

We included 27 patients. Median age at diagnosis was 18.3 years. Sekeletal tumors predominated (89%), mainly in the pelvis and lower limbs. Metastatic disease was present at diagnosis in 41%. Neoadjuvant chemotherapy was initiated after a median delay of 36 days post-biopsy, with a median duration of 22 weeks and a mean interval of 19.8 days. The mean relative dose intensity (RDI) was 70%, with only 11% of patients achieving an RDI >85%. A lower RDI was observed in adults (p=0.011) and metastatic stage patients (p=0.011). High-grade toxicities were reported in 89% of patients, primarily neutropenia. Good pathologic response was reported in 11 patients. After a median follow-up of 26 months, the median OS was 32 months, with 3-year OS and EFS rates of 36.9% and 19.9%, respectively. The relapse rate was 67%, with a median relapse time of 13 months.

**Conclusion:**

This study highlights the challenges of implementing the EE2012 protocol in our practice. Adapting the regimen could optimize induction, particularly for localized forms.

## Introduction

Ewing sarcoma (ES) is the second most common malignant bone tumor in patients under the age of 20. The incidence is one case per million, reaching a peak incidence of 10 cases per million in the second decade of life (1, 2).

Since the 1970s, the introduction of combined chemotherapy regimens has significantly improved prognosis, notably with VAC (Vincristine, Actinomycin D, Cyclophosphamide) and Doxorubicine (3–5). Subsequently, the addition of Ifosfamide and Etoposide (IE) alternating with VDC (Vincristine, Doxorubicin, Cyclophosphamide) improved therapeutic results in non-metastatic ES (6). The absence of new, effective cytotoxic molecules was the rationale for intensifying treatment. Dose escalation alone proved to have no significant benefit on survival. However, reducing the intervals from 21 to 14 days, under cover of growth factors, brought a significant benefit in terms of recurrence-free survival at 5 years without increasing toxicities (7).

This was the rationale for the international multicenter, randomized, controlled phase III “Euro-Ewing 2012” study that demonstrated the superiority, in terms of recurrence-free survival (RFS) and even overall survival (OS), of the interval-compressed VDC/IE protocol in comparison to the multi-agent protocol VIDE (Vincristine, Ifosfamide, Doxorubicine, Etoposide) previously used in European guidelines (8).

However, real-world feasibility of this intensified treatment, especially in low- and middle-income countries (LMICs), remains underreported. Furthermore, adult patients are underrepresented in major trials, with most data and practice being extrapolated from pediatric populations.

This study aims to assess the feasibility, tolerance, and report therapeutic outcomes of the ic-VDC/IE regimen in Tunisian patients treated at the Salah Azaiz Institute.

## Methods

### Study design and setting

A retrospective, observational cohort study was conducted at the Medical Oncology Department, Salah Azaiez Institute, Tunisia.

### Population

From January 2018 and April 2024, Tunisian patients with histologically confirmed Ewing’s sarcoma treated with the VDC/IE protocol were included.

### Diagnosis

Diagnosis was confirmed on microscopic examination. Histological diagnosis is based on the morphological appearance of small round cell proliferation, and positive CD99 membrane staining in immunohistochemistry. For further confirmation, the study of a t(11,22) translocation or genetic rearrangement has been performed.

### Staging

Staging workup included standard radiography and magnetic resonance imaging (MRI) of the affected limb and a PET-scan (Positron emission tomography-scan) or chest CT scan (computerized tomography), a bone scintigraphy, and a bone-marrow aspirate to determine disease dissemination. Cerebral-medullary MRI was performed depending on tumor location and presentation.

### Treatment protocol

Induction chemotherapy consisted of 5 cycles of VDC (Vincristine 2mg/m²/d x1day; maximal single dose of 2mg, Doxorubicine 35mg/m²/d x2days and Cyclophosphamide 1200mg/m²/d x1day) alternating with 4 cycles of IE (Ifosfamide 1800mg/m²/d x5days, Etoposide 100mg/m²/d x5days and MESNA) scheduled every 14 days. Chemotherapy was administered upon hematological recovery as per institutional guidelines: a recovery of neutrophil count to ≥1.5 x106/L, of platelet count to ≥100 x109/mm3 and of Hemoglobin to ≥8g/dL were required. Granulocyte colony-stimulating factor (G-CSF) support was delivered subcutaneously; (filgrastim 5µg/kg/d) starting from day 5 to day 10.

Induction therapy was followed by locoregional treatment which was surgery and/or radiotherapy pre-planned in a multidisciplinary tumor board (MTD). Consolidation therapy using IE (Ifosfamide 1800mg/m²/d x5days, Etoposide 100mg/m²/d x5days and MESNA) and VC (Vincristine 2mg/m²/d x1day; maximal single dose of 2mg and Cyclophosphamide 1200mg/m²/d x1day) was delivered after surgery, or concurrently with radiotherapy in inoperable tumors, independently of pathologic response due to local unavailability of High-dose chemotherapy and autologous stem cell transplant.

### Supportive care

Supportive care was delivered according to institutional regulations adapted to local resource availability. All patients were hospitalized for chemotherapy administration and monitored daily during treatment cycles. Haematological parameters were assessed prior to each cycle and once weekly during the inter-cycle period. Patients presented with grade 3 or higher neutropenia were systematically hospitalized and monitored for fever or signs of infection. In case of neutropenic fever, broad-spectrum antibiotic coverage was used. Taking into account the patient’s colonization as we as the department’s flora, Piperacillin-tazobactam was the first line treatment.

Prophylactic granulocyte colony-stimulating factor (G-CSF) was systematically administered following each VDC and IE cycle, starting at day +5 during 5 days, and in cases of prolonged use, was stopped 24 hours prior to chemotherapy. Red blood cell and platelet transfusions were performed according to institutional thresholds (haemoglobin <8 g/dL and platelet count <20 × 10^9^/L, or <50 × 10^9^/L in case of evidence of bleeding or invasive procedures).

Patients received antiemetic treatment using corticosteroids and ondansetron. Sufficient hydration with 2-3L/m²/day was administered during chemotherapy.

### Data collection and definitions

The data cut-off date for analysis was March 2024.

Clinical, pathological, radiological, and treatment data were collected, including doses, adverse events, objective response, surgical outcomes, histologic response, and survival outcomes.

The relative dose-intensity (RDI) was calculated. It is defined as the ratio of the dose received in mg/m² to the duration of treatment in weeks to the expected dose-intensity, expressed in percentage.

Occurrence of adverse events (AEs) was evaluated according to Common Terminology Criteria for Adverse Events (CTCAE) v5.0. Radiological response is defined according to RECIST criteria (Response Evaluation Criteria in Solid Tumors) v1.1.

Overall survival (OS) was defined as the time between the date of biopsy and the date of death or last follow-up. Event-free survival (EFS) was defined as the time between the start of treatment and the occurrence of progression, recurrence or death.

### Statistical analysis

Data analysis and results processing were carried out using Jamovi software. This was a descriptive study. Statistical tests used were the Chi2 test for comparison of frequencies and Student’s t-test for comparison of means, with a significance level of 5%. Fisher’s exact test was used to compare frequencies for small sample sizes. Correlations and associations between data were studied using Pearson’s bivariate correlation analysis and Student’s t-test for comparison of means. The Kaplan-Meier method was used to calculate 3-year OS, EFS and median survival. Given the limited sample size and number of events, only descriptive and univariate analyses were performed.

## Results

### Patients’ characteristics

A total of 27 patients were included. The mean age was 18.29 years ± 8.98 (median=18.3), with a sex ratio of 3.5 (21 male, 6 female). Sixty-three percent (63%) of patients were of urban origin. Pediatric patients predominated (18 versus 9 adults), and were mostly adolescents (n=12). The main presentations were pain, swelling and limping.

On average, the delay until first consult was 5.42 ± 4.87 months, with extremes ranging from 1 to 18 months. Patients from urban areas had on average a shorter delay (3.94 months) than those from rural areas (8.38 months) (p=0.032). This delay was shorter for patients under 18 years of age (a mean of 3.33 months versus 8.89 months, p=0.003).

Skeletal tumors were a majority (89%). Only three patients had extra-skeletal tumors (thigh soft tissue, kidney, infratemporal fossa). The main localization was the pelvis (n=6) and lower limb (n=13). Seven patients had a high tumor volume at presentation.

After work-up, 16 patients had localized Ewing’s sarcoma (59%) and 11 had metastatic disease (41%). Lung metastases accounted for most of metastatic sites (62%). Two patients had skip metastases.

*De-novo* metastatic disease was observed more frequently in the adult population (67%) than in the paediatric population (27%) (p=0.092).

### Feasibility and dose-intensity of induction chemotherapy

Induction chemotherapy with ic-VDC/IE was scheduled at intervals of 14 days. A total of 230 cycles were administered in a median duration of 22 weeks (range: 10–31 weeks) and at a median interval of 19.8 days (range: 16-27).

Prior to chemotherapy initiation, no patient had heart failure, and the mean LVEF was 67.19 ± 4.50% (ranging from 60 to 80%). Five patients initially had anaemia (21%). No abnormalities were found in the initial renal or hepatic workup, and no patient had initial hypoprotidemia.

Median delay from diagnosis to treatment initiation was 36 days (range 17 to 258) and was longer in adults (p=0.046). Most patients have completed all 9 induction cycles (n=23, 85%). All patients experienced treatment delay and re-scheduling because of insufficient hematologic recovery as per local institutional threshold. Fifteen patients required dose reductions.

Consequently, the mean RDI was 70 ± 10% and only three patients (11%) achieved RDI>85%. The RDI was higher during the IE sequence (an average of 72.3% for both Ifosfamide and Etoposide) than during the VDC protocol (68.1% for Doxorubicin and Cyclophosphamide). Dose reductions were more frequent in the VDC protocol than in the IE protocol.

Patients under 18 years of age received their treatment within shorter timeframes (**Table 1**). The RDI was higher in the pediatric population compared to the adult population. In fact, the mean RDI was 63.58% in adults versus 73.48% in children (p = 0.011). No adult achieved an RDI > 85%.

**Table 1:** Treatment timeframes according to age.

Treatment delays details	< 18 years N = 18	≥ 18 years N = 9	P
Median delay of treatment initiation	40 days	68,4 days	**0,046**
Median duration of treatment	33 weeks	58 weeks	**0,033**
Median duration of inter-cycle interval	18,33 days	20,38 days	**<0,001**
Median duration of treatment reschedules	9 weeks	12 weeks	**0,019**
Median delay from chemotherapy to surgery	4,5 weeks	5,5 weeks	0,64

Bold values indicate statistically significant.

RDI was not influenced by tumor location, whether axial or peripheral (p = 0.820), nor pelvic or extra-pelvic (p = 0.761). It was however significantly higher in patients with localized disease. **Table 2** summarizes the level of compliance to neoadjuvant treatment in the cohort.

**Table 2:** Compliance to treatment.

Compliance to treatment	N=27
Treatment completed without dose reduction	12 patients (44%)
Dose reduction during treatment	15 patients (56%)
Delay of chemotherapy cycles ≥ 7 days	24 patients (89%)
Mean number of delayed cycles per patient	4.33 cycles (range : 1-8)
Missed chemotherapy cycles	3 patients (11%)
Mean number of missed cycles per patient	2 cycles
Mean interval between end of chemotherapy and surgery	5.38 weeks (range: 2-12)

### Toxicity and tolerability

A total of 171 adverse events were reported during treatment. Twenty-four patients (89%) experienced toxicities of grade 3 or higher. The adverse events were mainly hematologic in nature. In three patients (11%), hematologic toxicity worsened during radiotherapy administered to the primary tumor. Neutropenia was the most frequently observed toxicity. It was recurrent in 48% of cases, with a median of 2 neutropenic episodes per patient. High-grade neutropenia occurred in 88.9% of patients (n=24). Among them, 48% (n=13) developed febrile neutropenia. Febrile neutropenia was recurrent in 38% of cases (n=5). In 74% of cases, the neutropenia occurred late, after the 10th day of the chemotherapy cycle.

A total of 29 chemotherapy cycles were complicated by thrombocytopenia. It was observed in 40% of patients (n=11), and was high grade in 21% (n=6). Platelet transfusion was required in one case of grade 4 thrombocytopenia.

Anemia complicated 38 chemotherapy cycles, affecting 48% of patients (n=13). It was high-grade in 29% of cases (n=11). Blood transfusions were required in 9 patients, with a mean of 2.44 red blood cell units transfused. Anemia was recurrent in 46% of patients (n=6).

Adults experienced more recurrent febrile neutropenia compared to children (p=0.018), as well as a higher incidence of recurrent thrombocytopenia (p = 0.063). Younger patients were more likely to develop more severe neutropenia (p = 0.025).

Other adverse events were also reported, including surgical or biopsy site infections (n=3), one case of extravasation of a vesicant cytotoxic drug, leading to tissue necrosis and substance loss and one case of acute kidney injury.

### Compliance to treatment

Surgery was delayed by more than 3 weeks in 11 patients (41%). In 6 of these cases, the delay was attributed to hematologic toxicities. The median cumulative duration of chemotherapy delays due to hematologic recovery was 37 days (range: 7 to 63 days), corresponding to an average of 2.45 days of delay per cycle.

More than half of the patients (n=15, 56%) required dose reductions. These were mainly due to treatment-related toxicity (n=12, 44%) and unavailability of G-CSF (n=3, 11%). Pediatric patients were more affected by dose reductions than adults (57% vs. 8%). The median time to first dose reduction was 24 weeks (range: 4 to 52 weeks). This corresponded to a mean reduction to 57% of the total target dose, with extremes ranging from 20% to 85.7%. Dose reductions were more frequent with the VDC triplet than with the Ifosfamide-Etoposide IE doublet.

Dose reductions due to toxicity were more common in children (64%) than in adults (30%) (p = 0.026). This strategy allowed treatment completion with less delay due to toxicity in pediatric patients (5.13 weeks in children vs. 8.22 weeks in adults on average, p = 0.019), thus the higher RDI in children.

### Tumor response and outcomes

At evaluation following induction chemotherapy, a partial response was observed in 23 patients (85%), stable disease in 3 patients (11%), and disease progression in 1 patient (4%). Disease progression was noted at the time of locoregional treatment in two patients who initially had responded.

Patients having received ≥70% of RDI were more likely to have resectable tumors (p=0.036). Surgery was performed at a median interval of 4.5 weeks after the completion of induction chemotherapy, with a range of 2 to 12 weeks. This interval exceeded 3 weeks in 11 patients, including 3 of the 4 patients treated during the COVID-19 pandemic. No postoperative complications were reported.

Good pathologic response was reported in 11 patients. Nine had a complete pathological response.

Pathologic response was correlated to the RDI during neoadjuvant treatment (p=0.042) and to the delay of surgery (p=0.034) (**Table 3**). A significant association between histological response and relative dose intensity was observed in subgroups defined by age. Dose reductions did not have a significant impact on histological therapeutic effect (p = 0.98).

**Table 3:** Factors impacting pathologic response.

Factors	Good response	Poor response	P
RDI of induction CT (means)	72,92 ± 7,73%	67,83%	**0,042**
Induction CT duration (means)	21,33 ± 2,67 weeks	23,25 ± 2,63 weeks	0,186
Chemotherapy-Surgery Delay (means)	4,5 ± 2,3 weeks	8 ± 3,26 weeks	**0,034**

CT, Chemotherapy; RDI, Relative dose-intensity.

Bold values indicate statistically significant.

### Survival

**Figure 1:**
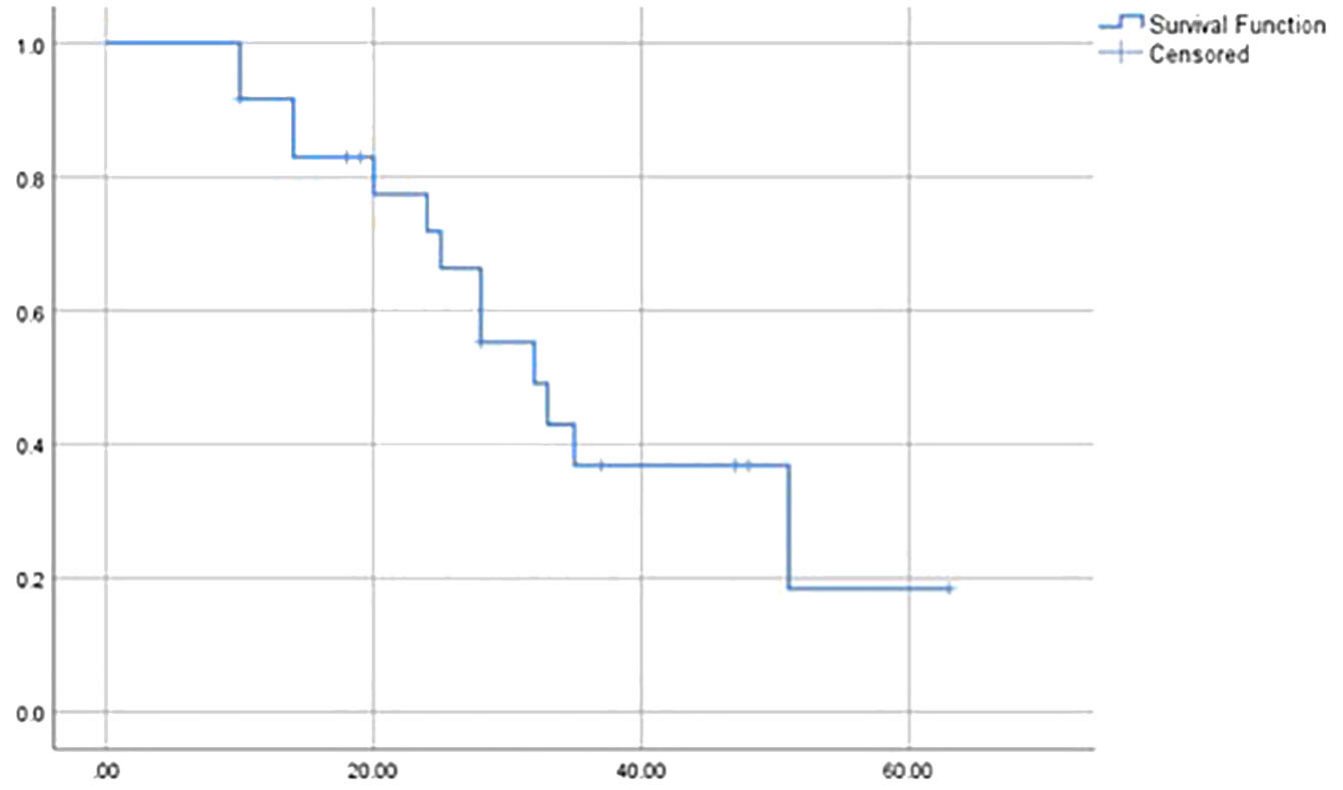
Kaplan-Meier estimate of overall survival (months).

After a median follow-up of 26 months (range 11-62), the median overall survival (OS) was 32 months (range: 10 to 63 months) and 3-year-OS was 36.9% (**Figure 1**). OS data remains immature given the short follow-up. Twelve patients (44%) were alive at the time of analysis. Eight had no evidence of disease and four are undergoing treatment for relapse. The main cause of death was disease progression (93%). One death was attributed to toxicity from second-line treatment.

**Figure 2:**
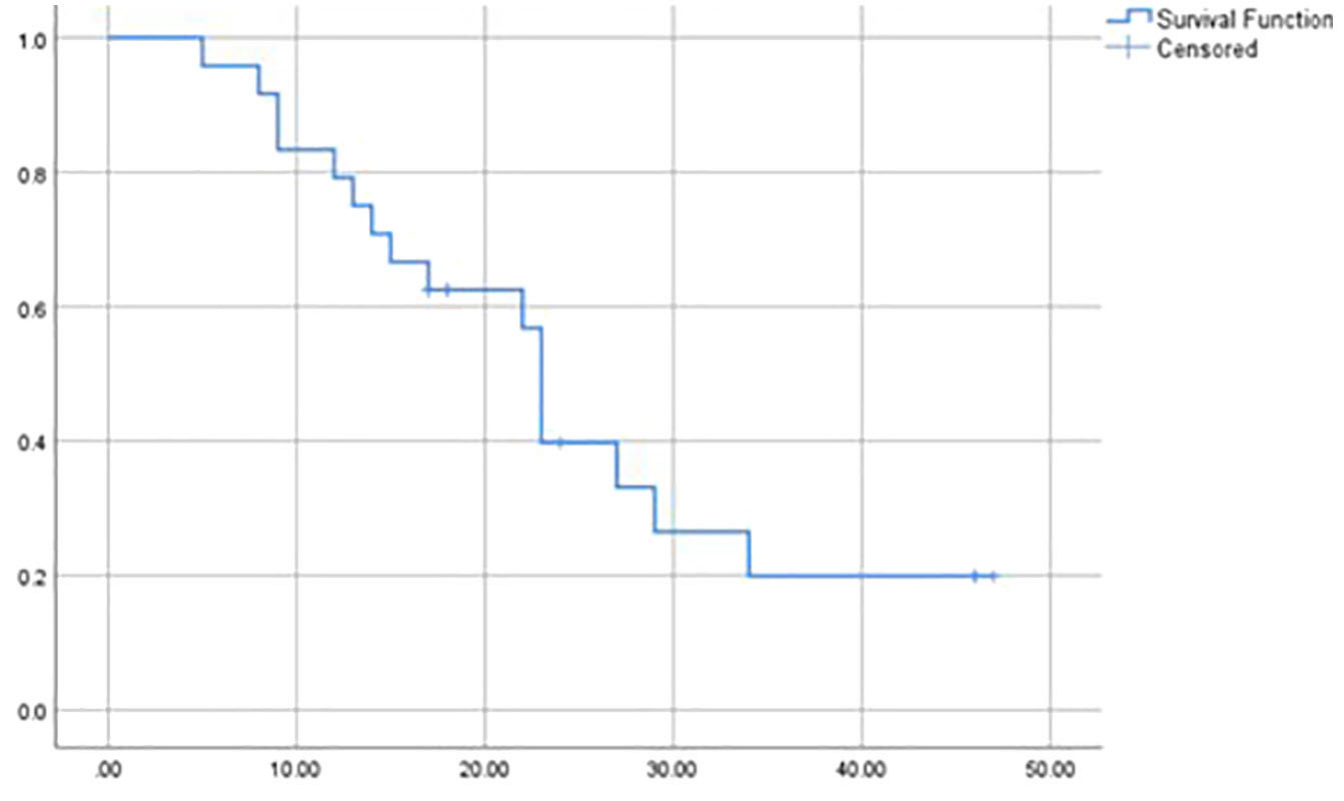
Kaplan-Meier estimate of event free survival (months).

The relapse rate was 67% (n=18). The main sites of metastatic relapse were pulmonary (n=14) and nodal (n=2). The median time from completion of therapy to relapse was 13 months (range: 3-38). The median event-free survival (EFS) was 23 months (range: 5-47). Event-free survival rates were 79.2% at 1 year, 39.8% at 2 years, and 19.9% at 3 years (**Figure 2**).

## Discussion

### Population

Ewing’s sarcoma is the second most common childhood malignant bone tumor after osteosarcoma. The incidence rate is estimated at 7 to 8 cases per million in Europe and 1 to 2 cases per million in North America (1, 2). Peak incidence occurs during adolescence. According to large retrospective studies, the median age of onset of Ewing’s Sarcoma is estimated at 15 years (3–5).

The various retrospective studies show a slight male predominance (between 55 and 67%), with a sex ratio of 1.47 (6, 7). The adult population is rarely affected. Indeed, subjects over the age of 40 account for less than 20% of diagnosed cases of Ewing’s sarcoma, all sites combined (9).

In line with international, national and local data, our population included more adolescents (n=12, 44%) than children and adults. The mean age at diagnosis was 18.29 years, with a median of 15 years. On the other hand, our population showed a marked male predominance, with 78% male subjects and a sex ratio of 3.5. This difference could be attributed to the small size of our study and its retrospective nature.

### Pre-therapeutic imperatives

According to the “Euro-Ewing 2012” protocol, VDC and IE courses are administered upon hematological recovery defined as an ANC count ≥0.75x10^9^/L and platelet count ≥75x10^9^/L, at intervals of 14 ± 3 days.

In our institution, a more conservative hematological threshold is applied before chemotherapy re-administration (ANC ≥1.5 × 10^9^/L, platelets ≥100 × 10^9^/L, and hemoglobin ≥8 g/dL). This resulted in a cumulative treatment delay of 34 days on average per patient compared with the theoretical protocol schedule.

These adapted thresholds reflect local clinical constraints and patient-related vulnerabilities that differ substantially from the conditions in which the Euro-Ewing 2012 protocol was originally developed. Our patient population is characterized by frequent long-distance travel to the treatment center, limited access to emergency care, suboptimal socio-economic conditions, and heterogeneous educational backgrounds. In this context, post-chemotherapy complications—particularly febrile neutropenia and bleeding—may be associated with delayed presentation and limited access to timely supportive care.

Furthermore, resource constraints impact the availability of immediate hospitalization, transfusion support, and outpatient monitoring in the event of acute toxicity. As a result, early re-administration of chemotherapy at lower hematological thresholds may expose patients to an unacceptable risk of severe infection, hemorrhage, and treatment-related mortality.

In fact, in our cohort, infectious complications and transfusion requirements were frequent during early cycles, reinforcing the need for cautious re-administration of chemotherapy. Several patients required prolonged hospitalization for febrile neutropenia, and access to emergency services was not uniformly available in their home regions. These observations further supported the adoption of higher hematological recovery thresholds.

The decision to use higher hematological recovery thresholds was therefore driven by a strategy of clinical vigilance and patient safety, prioritizing the prevention of life-threatening complications over strict adherence to dose-dense scheduling. This pragmatic adaptation represents a compromise between protocol intensity and real-world feasibility in a low-resource setting.

### Intensification of induction chemotherapy

In the absence of new, effective cytotoxic molecules, chemotherapy is intensified out of necessity, with a view to optimizing the treatment of Ewing’s sarcoma. Some studies have explored the feasibility of dose escalation, while others have hypothesized a reduction in the inter-cycle intervals.

While dose escalation alone did not provide any survival benefit, in addition to an increase in acute toxicities; the reduction of intervals from 21 (VDC/IE) to 14 days (ic-VDC/IE) revealed a significant advantage. In fact, 10-year EFS increased from 61% to 70% for localized ES (p=0.03) and the 10-year-OS increased from 69% with 3-weekly VDC/IE to 76% with ic-VDC/IE (p=0.04) (10).

The administration of chemotherapy every 14 days, feasible under growth factor support (G-CSF), had not resulted in increased toxicities and this regimen has been adopted as the therapeutic standard in the US (11, 12).

The ic-VDC/IE protocol also demonstrated superiority in terms of recurrence-free survival and overall survival over the European standard of care (VIDE) in the “Euro-Ewing 2012” clinical trial for localized ES (13, 14). In fact, the 3-year relapse-free survival (RFS) rate increased from 58% in the VIDE arm to 69% in the VDC/IE-ic arm (HR 0.66; IC95% [0.49-0.9]). The 3-year OS rate increased from 74% to 82% (HR 0.64; CI95% [0.42-0.96]) (13).

By analogy, this intensified regimen has also been validated for patients in the adult population (15, 16). The benefit of shorter intervals for metastatic Ewing’s sarcoma was not significant. Thus, the ic-VDC/IE protocol has been adopted as the therapeutic standard in Tunisia for pediatric and adult Ewing sarcomas since 2018.

### Feasibility of interval-compressed chemotherapy

Since the implementation of G-CSF, chemotherapy treatment intervals have been shortened in order to intensify treatment. This made it possible to increase the delivered dose-intensity (12). As primary prophylaxis, growth factors have been shown to maintain an optimal dose-intensity in patients undergoing chemotherapy for potentially curable tumors (17, 18). Prospective studies of intensified (dose-dense) chemotherapy have demonstrated its feasibility under G-CSF coverage, without any increase in hematological toxicity (19, 20).

The feasibility of the ic-VDC/IE protocol was first demonstrated in a pilot study in which G-CSF from D5 of the course of treatment enabled inter-cures to be reduced to a median of 16 days. With this regimen, the dose-intensity was multiplied by 1.27 (12). The postponement rate varied from 18% to 64% across the different studies (21, 22).

The AEWS0031 trial is the main clinical trial to directly compare the two treatment regimens with VDC/IE (standard arm) versus ic-VDC/IE (experimental arm). In the experimental arm, the mean duration of intervals was 17.29 days, and was similar in children and adults (17.31 days and 17.16 days, respectively) (11). RDI has not been reported.

In a retrospective series reporting RDI data in patients treated for Ewing’s sarcoma, the rate of patients with an acceptable RDI (≥85%) did not exceed 60%, with an estimated median RDI of 87% (22).

These results suggest that treatment with an intensified protocol (ic-VDC/IE) is hardly feasible, even under G-CSF. Maintaining a high RDI is only achieved in almost half of treated patients. Most patients required frequent rescheduling of cycles.

According to a large retrospective multicenter analytical study including 66 adults and 41 children treated for Ewing’s sarcoma, the pediatric population received ic-VDC/IE more frequently (76%) than adolescents and young adults (AYA) (31%) (p<0.001). Children also appeared to start treatment more quickly than AYA, and had a better chance of achieving satisfactory RDI during induction therapy (22, 23).

In our study, most patients had completed their induction treatment (89%), with a mean interval duration of 19.8 days in the induction phase. All patients required a rescheduling of treatment at least once. Consequently, only 48% of patients had achieved an RDI of 70% during neoadjuvant CT. Even fewer patients reached the RDI threshold of 85% (11%).

This reflects the difficulty of maintaining an optimal dose-intensity in our patients. The main reason for postponing courses of treatment was failure to achieve the required hematological recovery (ANC≥1.5x109/L, a Platelet count ≥100x109/L and a Hb count ≥8g/dL).

This postponement of cycles, and, consequently, the reduction in dose-intensity received, is due to a pre-therapeutic requirement greater than that indicated by the protocol itself. This is due to precautions dictated by local practices and experience acquired in the face of an increased risk of toxicity in our socio-economic context.

Our findings are consistent with reports from other LMIC settings, where the delivery of dose-dense chemotherapy is frequently compromised by logistical and supportive care constraints. Treatment delays and dose modifications are commonly observed and may impact outcomes. In this context, our study illustrates the challenges of implementing the EE2012 protocol outside high-resource settings and underscores the need for adapted strategies (24, 25).

In line with the results of larger studies, the mean duration of inter-treatment in the induction phase was longer in adults (22.42 days) than in children (18.2 days) in our population (p<0.001).

However, the cumulative delay for hematological non-recovery was more or less the same in both groups (an average of 35 days in children and 32 days in adults). On the other hand, dose reductions were more frequently prescribed in the pediatric population. This reflects the determination to maintain a high RDI more aggressively in children than in adults, by reducing doses rather than postponing the course of treatment.

In addition, children in our population actually started their induction chemotherapy sooner than adults. The reasons for this difference cannot be deduced from retrospective data, but are most likely related to a prioritization of children in the hospital and oncology care setting.

### Prognostic value of the relative dose-intensity

*In vitro*, a direct correlation has been demonstrated between the concentration of many cytotoxic agents and tumor lysis, and inversely with the rate of tumor growth (26). Similarly, a relationship between dose and response has been demonstrated *in-vivo* in various animal models. One study suggested that as little as a 20% reduction in total dose reduced the chance of cure by 50% (27).

In human patients, clinical data confirm the existence of a dose-response relation in cancer patients, regardless of stage of the disease. It has been established that the RDI of chemotherapy correlates with oncological outcomes, for all tumor types, both in the adjuvant and metastatic setting (17, 18). In the metastatic phase, RDI is associated with a better objective response rate and progression-free survival. In the early phase, reducing the RDI is associated with a deterioration in therapeutic results (18, 28, 29).

RDI has been recognized as a major prognostic factor in sarcomas, where reduced RDI, through reduced treatment doses or treatment delays, was associated with less favorable therapeutic outcomes (30–33). But RDI data in Ewing’s sarcoma treatment protocols are poorly reported. A cohort study of 81 Chinese ES patients receiving VDC/IE (at 21-day interval) revealed that a chemotherapy delay of more than three days was associated with poorer survival, in multivariate analysis, underlining the importance of dose-intensity optimization (34).

In our population, RDI was one of the predictors of histological response (p=0.042). The impact of RDI on OS and EFS was not feasible given the sample size, but we can extrapolate its prognostic value from its impact on pathologic response, an established surrogate for survival outcomes in Ewing’s sarcoma.

### Toxicity of intensified chemotherapy

Maintaining an acceptable RDI is directly linked to the tolerance and management of chemotherapy toxicity. This represents a challenge in daily practice, especially in the context of intensified treatment. Across all subgroups analyzed, the predominant toxicity is haematological. It is the leading cause of dose reduction (34–36). According to the Children’s Oncology Group report, the most frequent toxicity of the ic-VDC/IE protocol was infection with or without neutropenia. The occurrence of neutropenia varies from 30 to 60% of patients, depending on the study (24, 37). The occurrence of anaemia or thrombocytopenia requiring transfusion ranged from 5 to 33% and 15 to 62%, respectively (24, 37). Other toxicities such as mucositis and urinary tract infections are also frequently reported. Treatment-related gonadal toxicity varies from 3% to 18% depending on the series, and is higher in men than in women (38–40).

According to the different cohorts, dose reductions during intensified chemotherapy were necessary in 29% to 40% of patients. Dose reduction was more common with the Doxorubicin-Cyclophosphamide doublet (49%) than with the Ifosfamide-Etoposide doublet (33%) (41).

In practice, clinicians often fear that the intensified regimen will lead to increased haematological toxicities and treatment delays, particularly in adults, since tolerability is not clearly described in the adult population. Indeed, the adult population is not widely included in flagship clinical trials investigating the treatment of Ewing’s Sarcoma. Patients aged over 18 represented only 12% of the population included in the AEWS0031 trial that received ic-VDC/IE (10).

Two studies have infirmed the relationship between age and the occurrence of toxicities, suggesting the feasibility and safety of this intensification in adults (16, 42). According to a meta-analysis including 4838 patients followed for sarcoma, AYAs have statistically fewer high-grade toxicities (43). The authors also noted higher toxicity in women than in men. Although concerns remain about the tolerability of AYAs in the face of intensive treatment compared with children, a safety analysis of the EURO-E.W.I.N.G.99 study, which included 224 patients aged between 19 and 50, revealed that the frequency of severe adverse events tended to decrease with age (31). However, it remains unclear whether this difference in frequency is linked to a difference in tolerance, dose adaptation or compliance with treatment (31, 44). In view of these facts, intensification of treatment by reducing the duration of intervals remains recommended in adults with localized disease, as it has proved its worth in terms of therapeutic results compared with the conventional regimen (45).

In our study, as in the literature, the most frequent toxicity was hematological toxicity and, in particular, neutropenia. Almost half the patients (48%) had developed febrile neutropenia despite primary prophylaxis with G-CSF. A large proportion of patients (89%) experienced high-grade toxicities requiring inpatient management, with hospital stays ranging from 2 to 32 days. However, all these toxicities were manageable with specific and symptomatic treatment, and none resulted in toxic death.

Conclusions as to the factors explaining this excess risk of toxicity in our population cannot be drawn from a small-scale study. However, these data do explain the increased vigilance and precaution in administering chemotherapy courses from a higher hematological recovery threshold. These high toxicities also explain the more frequent recourse to dose reduction (44%) compared with the various international series. These dose reductions are sometimes carried out very early (as early as the 4th week of treatment) in order to maintain a balanced benefit-risk ratio.

Finally, our study, in line with larger studies, invalidates the relationship between age and treatment tolerance (p=0.703), apart from the depth of neutropenia in the event of its occurrence.

### Choice of consolidation chemotherapy

The international Euro-E.W.I.N.G.99 and Ewing 2008 trials compared intensification with high-dose chemotherapy (HDCT) with Busulfan-Melphalan followed by autologous hematopoietic stem cell transplantation (ASCT) to standard chemotherapy with VAI (Vincristine, Actinomycin D and Ifosfamide), in consolidation after induction chemotherapy with VIDE and surgery (46).

The aim of both trials was to evaluate the efficacy and safety of high-dose chemotherapy in patients in the high-risk group (Randomization R2loc) or those with exclusive pleuropulmonary metastases (Randomization R2Pulm), aged under 50 years.

HDCT was found to significantly improve overall survival and recurrence-free survival in localized Ewing’s sarcoma classified as high-risk (R2loc randomization), but not in metastatic Ewing’s sarcoma (R2Pulm randomization). Despite the increase in acute toxicities observed, HDCT has thus become the therapeutic standard in consolidation for localized Ewing sarcomas in the high-risk group treated with VIDE induction chemotherapy (46–48). According to real-world data, this intensification is more frequently chosen and administered in children than in adults (23).

Intensification with Busulfan and Melphalan followed by ASCT is currently unavailable for Ewing’s sarcoma in Tunisia. This is why all our patients had received standard consolidation chemotherapy with VC/IE, despite the fact that this represents a loss of chance for our patients.

### Survival

Short-interval chemotherapy provides a significant benefit in terms of overall survival and event-free survival for localized ES. Despite the dose-intensity increase, the risk of secondary neoplasia does not appear to be increased by short-interval chemotherapy (10). The 5-year overall survival of patients diagnosed with non-metastatic Ewing’s sarcoma ranges from 65 to 80%. Recurrence-free survival at 5 years reaches 78% for localized ES. In subgroup analysis, adulthood is associated with poorer overall survival. This falls from 75% in children to 58% in adults at 5 years (49).

The latest Eurocare-5 program showed that adolescents with ES still have poorer overall survival than children, with no improvement over recent decades (50, 51).

The AEWS1031 trial described a higher risk of death in the adult population than in the paediatric population (4). It is important to remember that axial or pelvic localization seems to be more frequent in the adult population, with early metastatic disease and higher tumor volume (52). However, according to several studies across different ethnic groups (American, European, Japanese, Nordic, Irish), adult patients as well as adolescents still have a lower survival rate than children, with an estimated 18-20% loss of survival rate at 5 years, irrespective of the location of the primary tumor, its skeletal or extra-skeletal origin and the stage of the disease (51–56).

As suggested, adult patients tended to be less likely to receive a RDI ≥85% (22). It is likely that the disappointing therapeutic results in adult patients are related to undertreatment. Indeed, in a cohort of 81 patients, Zhang et al. concluded that a minimum of 12 cycles of VDC/IE completed without deferrals was a major prognostic factor for adult patients (34). Another study lowered this threshold to a minimum of 10 cycles (35).

However, this intensified regimen was not associated with better survival in the adult subgroup with localized disease (23). One study suggests that in the context of on-time completion of treatment without dose reduction, with a RDI ≥85%, no difference in survival is seen between adult and pediatric patients (30). In another study, among patients under 35 years of age, the only predictors of overall survival were RDI and histological response to neoadjuvant chemotherapy (30). This reflects the importance of the prognostic value of relative dose-intensity rather than age itself.

Metastatic disease remains the major challenge in the treatment of children and young adults with Ewing’s sarcoma, despite the good results of ic-VDC/IE in localized sarcomas. Patients with metastatic sarcomas often have a good initial response to chemotherapy, but this is usually followed by progression within two years (57). The 5-year overall survival rate falls from 70% to 30% in the metastatic setting, or even 20% in the adult population (53).

In our population, the median OS was 32 months, and the relapse rate was 67%, with a median recurrence-free survival of 23 months. However, these results fall far short of the expected therapeutic outcomes, given the data from large therapeutic trials, the small sample size and the inadequate RDI.

Our patients rarely received treatment with a RDI exceeding 85%, a value considered to be the threshold of acceptability across the different series. This reflects the challenge and public health problem that Ewing’s sarcoma still represents, and its intensified treatment despite recent therapeutic advances.

The median follow-up time of 26 months nevertheless represents an inadequate follow-up in terms of survival. Survival outcomes should be interpreted with caution given the relatively short follow-up. It is important to remember that, on the one hand, a significant proportion of our population (41%) is diagnosed at the metastatic stage; on the other hand, localized Ewing’s sarcomas in the high-risk group do not benefit from intensified treatment, which could improve their survival.

### Perspectives

Chemotherapy with ic-VDC/IE has demonstrated its efficacy in prospective multi-center clinical trials. It offers a number of advantages, including:

- Better local control, as evidenced by improved rates of operability, complete surgery and major histological response.

- Better survival, confirmed in prospective multicenter studies in Europe and the United States.

- The possibility of completing treatment in a shorter period of time, which is especially reassuring for the patient.

The main disadvantage of interval-compressed chemotherapy is the shortened rest time between chemotherapy cycles. However, toxicity is no greater than with conventional chemotherapy.

High-grade toxicities remain frequent despite G-CSF support and even with a high hematological recovery threshold required prior to treatment. However, these toxicities remain manageable. Thus, it is preferable to consider dose reduction rather than delaying treatment when these side effects occur (57).

Early initiation of G-CSF prophylaxis and a longer duration of injections could be solutions for maintaining an acceptable dose-intensity. Adapting the treatment dose to the patient’s weight and providing therapeutic education could help avoid excessive and deleterious treatment delays, especially in the case of localized disease eligible for surgery. With proper patient selection and armed monitoring, including telephone follow-up, a lower threshold of ANC and platelet count could be accepted prior to chemotherapy.

For patients in the high-risk localized disease subgroup, the benefits of HDCT have been proven. To improve therapeutic outcomes, the development of a consensus definition of the localized high-risk group, incorporating clinical and molecular characteristics, will enable the stratification of these patients to be harmonized internationally. Improved global access to HDCT and ASCT for these patients is essential.

Innovation is currently a necessity in order to improve therapeutic outcomes for patients at the metastatic stage and in cases of rarely curable recurrence. Clinical trials are underway to evaluate the efficacy of various treatments for metastatic sarcoma, such as Ganitumab, tyrosine kinase inhibitors like anti insulin-like growth factor-1 receptor (58). Immunotherapy, such as Pembrolizumab or Nivolumab (with or without Ipilimumab), is not very effective in the treatment of Ewing’s sarcoma.

## Conclusions

Despite its retrospective nature and small sample size, our study is one of the few real-life studies reporting the feasibility of the “Euro-Ewing 2012” protocol. After exhaustive analysis of a total of 230 induction chemotherapy cycles, we were able to conclude that this intensified was not feasible in our population.

In patients with localized disease, earlier initiation of growth factors and dose reduction rather than treatment postponement could guarantee high-dose-intensity induction chemotherapy.

In patients with metastatic disease, the conventional 21-day interval VDC/IE protocol appears to be the most suitable for achieving a response while preserving the patient’s quality of life.

Inclusion of the adult population in future prospective multi-center clinical trials will help minimize disparities in therapeutic outcomes. Establishing international data networks that include patients from low- and middle-income settings would help reduce the gap in cancer care.

## Data Availability

The original contributions presented in the study are included in the article/supplementary material. Further inquiries can be directed to the corresponding author.
